# Scenario analysis for programmatic tuberculosis control in Bangladesh: a mathematical modelling study

**DOI:** 10.1038/s41598-021-83768-y

**Published:** 2021-02-23

**Authors:** Md Abdul Kuddus, Michael T. Meehan, Md. Abu Sayem, Emma S. McBryde

**Affiliations:** 1 Australian Institute of Tropical Health and Medicine, James Cook University, Townsville, QLD Australia; 2 College of Medicine and Dentistry, James Cook University, Townsville, QLD Australia; 3 Department of Mathematics, University of Rajshahi, Rajshahi, 6205 Bangladesh; 4 Divisional Tuberculosis Expert, Khulna Division, National Tuberculosis Control Program (NTP), Directorate General of Health Service (DGHS), Dhaka, Bangladesh

**Keywords:** Computational biology and bioinformatics, Diseases, Mathematics and computing

## Abstract

Tuberculosis (TB) is a major public health problem in Bangladesh. Although the National TB control program of Bangladesh is implementing a comprehensive expansion of TB control strategies, logistical challenges exist, and there is significant uncertainty concerning the disease burden. Mathematical modelling of TB is considered one of the most effective ways to understand the dynamics of infection transmission and allows quantification of parameters in different settings, including Bangladesh. In this study, we present a two-strain mathematical modelling framework to explore the dynamics of drug-susceptible (DS) and multidrug-resistant (MDR) TB in Bangladesh. We calibrated the model using DS and MDR-TB annual incidence data from Bangladesh from years 2001 to 2015. Further, we performed a sensitivity analysis of the model parameters and found that the contact rate of both strains had the largest influence on the basic reproduction numbers ${\text{R}}_{{0{\text{s}}}}$ and ${\text{R}}_{{0{\text{m}}}}$ of DS and MDR-TB, respectively. Increasingly powerful intervention strategies were developed, with realistic impact and coverage determined with the help of local staff. We simulated for the period from 2020 to 2035. Here, we projected the DS and MDR-TB burden (as measured by the number of incident cases and mortality) under a range of intervention scenarios to determine which of these scenario is the most effective at reducing burden. Of the single-intervention strategies, enhanced case detection is the most effective and prompt in reducing DS and MDR-TB incidence and mortality in Bangladesh and that with GeneXpert testing was also highly effective in decreasing the burden of MDR-TB. Our findings also suggest combining additional interventions simultaneously leads to greater effectiveness, particularly for MDR-TB, which we estimate requires a modest investment to substantially reduce, whereas DS-TB requires a strong sustained investment.

## Introduction

Tuberculosis (TB) kills more people each year than any other infectious disease, including HIV and malaria, making it one of the primary global health problems^1^. In 2019, the WHO estimated there were approximately 10.0 million new cases of TB, and 1.2 million died from TB disease. Most of the estimated cases in 2019 occurred in Asia (44%) and Africa (24%) and 87% of tuberculosis deaths occurred in low and middle-income countries^1^. A significant proportion of cases occurred in the Western Pacific region (18%), with the Eastern Mediterranean region (8%), the European region (3%) and the Americas region (3%) also contributing small proportions^1^. Worldwide there is an imbalance in case notification between males (5.6 million new cases in 2010) and females (3.2 million new cases in 2010), which may have many causes, including missed cases. Childhood TB is often missed, as classical diagnosis with sputum smear is insensitive^2^.

In recent years, antibiotic resistance to the most effective treatments (first-line combination therapies) has emerged and spread. This has led to a decline in the efficacy of antibiotics used to treat TB, with drug-resistant (DR) TB patients experiencing much higher failure rates. The treatment regime for DR patients is more expensive than it is for DS patients and the diagnosis for DR patients is difficult—especially in low- and middle-income countries. As a result, the DR- TB mortality rate is much higher than that of DS-TB^3^.

There are two ways DR-TB can develop: one is called amplification; and the other is primary transmission. Amplification develops mainly through naturally-occurring mutations and inappropriate treatment^4^. Once preliminary resistance has been established, acquisition of further resistance to supplementary drugs becomes more likely as treatment with standard regimens may be suboptimal.

Primary transmission is when an individual with DR-TB directly infects a susceptible individual. Primary transmission was initially not expected to contribute significantly to the overall DR-TB burden due to the reduced fitness/transmissibility of DR organisms^5^. However, subsequent evolution and compensatory mutations can restore fitness in the absence or presence of antimicrobials^6^. The WHO recommends that timely identification of DR-TB and adequate treatment regimens with second-line drugs administered early in the course of the disease are essential to stop primary transmission^1^.

Currently, multidrug-resistant (MDR) TB is emerging as the greatest threat to TB control globally^1^. MDR-TB is defined as TB that is resistant to both isoniazid and rifampicin (the two most effective and commonly used first-line drugs) with or without resistance to additional first-line drugs. Cohort studies within programs of TB treatment estimate that approximately 1% of a treated population who begin with a susceptible organism will develop MDR-TB^7^. Once these new MDR-TB cases have emerged in the community, they are capable of spreading the infection through primary transmission, further contributing to the growing pool of MDR cases. Therefore, control of MDR-TB requires the prevention of both acquired drug resistance and subsequent transmission as well as effective diagnosis and treatment for those cases that do emerge^8^.

The highest burden of TB is not surprisingly in regions where health systems are weak. In 2015, the WHO recognised 22 high burden countries according to their actual number of TB cases^9^. Among these is Bangladesh, a country where poverty, high population densities, and malnutrition are commonplace, creating a favorable environment for TB outbreaks. Furthermore, TB treatment compliance is poor in Bangladesh—presumably as a result of the extensive period of therapy—leading to a rise in the number of TB cases^10^. Each year it is estimated that 70,000 people die of TB and 300,000 new cases appear in Bangladesh^2^. Prior to 2014, the case notification rates per 100,000 population were 68 and 122 for new smear-positive cases (i.e. cases that are usually more infectious and have a higher mortality) and all forms of TB cases respectively, but by the end of 2014 the number of all types of TB cases had increased, with a substantial increase in the number of extra-pulmonary cases due to better case detection^10^.

In Bangladesh, the NTP aims to sustain the global targets of achieving at least 70% case detection and 85% treatment success among new smear-positive TB cases for the whole country. The overall progress in case finding was slow and steady until 2016 reaching a case notification rate 138 per 100,000 population. The NTP achieved its objectives in effective treatment, case detection and overall management through partnership with other public and private health care providers, engaging all care providers (GO-NGOs) and making available free diagnostic and treatment support, particularly for DS-TB^11^. By 2003, the treatment success rate of this program reached the targeted 85% and has been maintained at 90% since 2005. In 2013, the program successfully treated 94% of notified new smear-positive cases and the case detection rate was about 58%^12^.

Mathematical models can improve our understanding of the epidemiology of TB as well as those components that are significant to TB diagnosis, treatment, and control^13–18^. Mathematical models are also useful for simulating different interventions and “what if” scenarios that would otherwise be infeasible in clinical trials due to ethical/logistical/practical concerns. These tools inspire researchers to eliminate trial and error methods and direct them towards rational, evidence-based decisions^19–24^. For example, Okuonghae and Ikhimwin (2015) developed a realistic compartmental transmission dynamic TB model^25^. According to the awareness level of the population, Okuonghae and Ikhimwin model divided susceptible persons into two groups; the high risk group (low level of awareness), and low risk group (high level of awareness), and incorporated an active case finding parameter. This study showed that TB treatment alone may not significantly reduce TB burden at the community level but if we take two or more interventions together, such as treatment, awareness and active case finding, then it may be possible to reduce TB burden. Kim et al.^22^ developed a mathematical model for TB with exogeneous reinfection and examined the current situation of active TB incidence in Korea. The results showed that case detection was the most important intervention for decreasing active TB cases and demonstrated that treatment or case discovery alone will not dramatically affect the decline in active TB incidence. Okuonghae and Omosigho^26^ developed a qualitative and quantitative approach to a transmission dynamic TB mathematical model in Benin City, Nigeria. This study showed that developing a TB awareness program and also increasing the active cough identification rate decreased the TB burden in the population, ultimately bringing down the basic reproduction number under unity. Furthermore, mutually raising the TB consciousness program and the raising or lowering of the cost of treatment in recognized cases can also decrease the basic reproduction number below unity^26^.

In this study, we considered a two-strain TB model to describe the transmission dynamics of DS and MDR-TB in Bangladesh. The model is calibrated to the Bangladesh demographic and DS and MDR-TB annual incidence data from years 2001 to 2015 to estimate the key transmission and fitness cost parameters. Multiple intervention strategies were considered to explore the impact of each on its own and when combined on DS and MDR-TB incidence and mortality. This study depicts Bangladesh-specific elimination policies and describes the results of different levels of investment in future on TB control: business as usual, modest investment (low and higher), strong investment for five years and sustained investment.

## Results

### Estimation of model parameters

**Figure 1:**
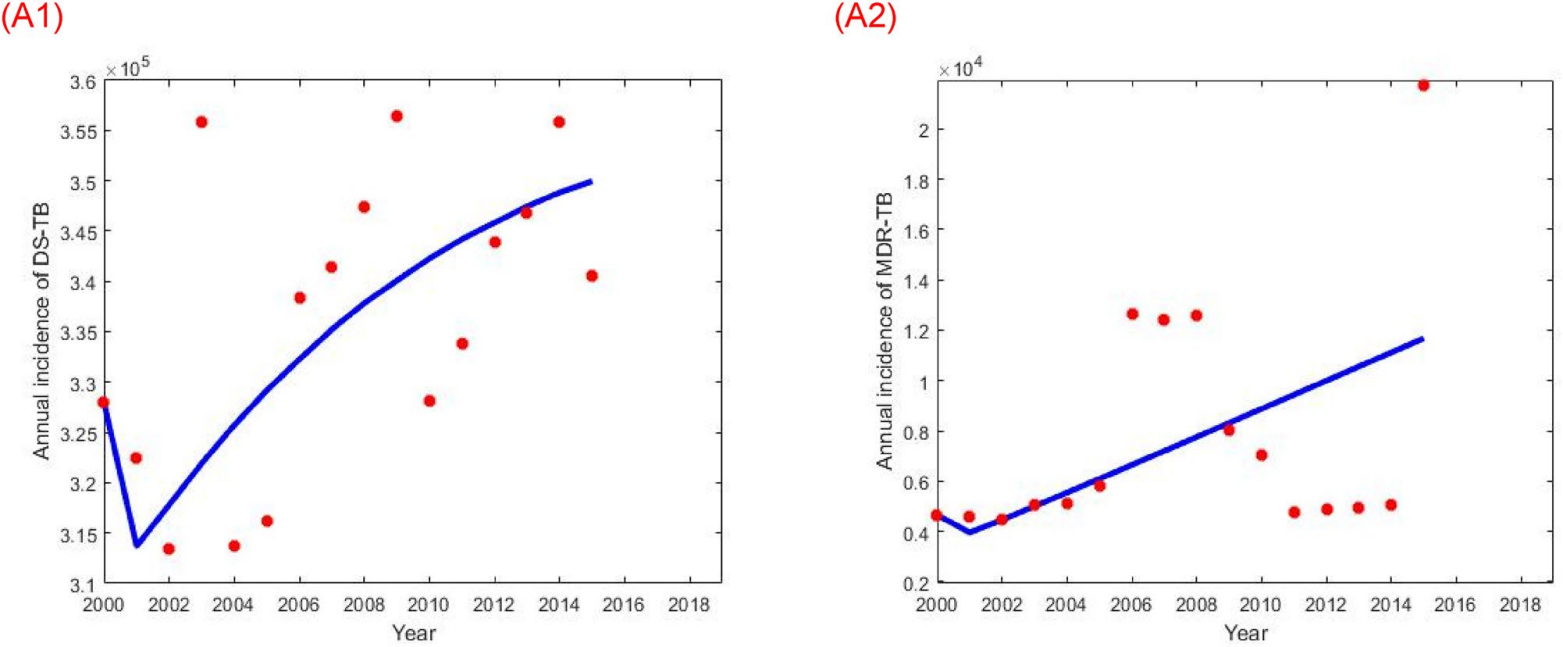
Reported Bangladesh TB annual incidence data as estimated by the WHO (red dots) and the corresponding best fit (blue solid curve): (left) drug-susceptible (DS) TB and (right) multidrug-resistant (MDR) TB.

In this section, we estimated the model parameters based on DS and MDR-TB annual incidence data taken from World Health Organization (WHO) report from 2001 to 2015. In order to fully parameterise the TB model (2), we obtained some of the parameter values from the literature (Table 1), and the rest of the model parameters were estimated using the least-squares fitting method which provides a better fit of the model solution to the annual DS and MDR incidence data (Fig. 1). The objective function used in the parameter estimation is as follows

$$\begin{aligned} {\hat{\uptheta }} & = {\text{argmin}}\mathop \sum \limits_{{{\text{i}} = 1}}^{{\text{n}}} \left( {\mathop \smallint \limits_{{{\text{t}}_{{\text{i}}} }}^{{{\text{t}}_{{\text{i}}} + 1}} \left( {{\upalpha {\text{L}}}_{{\text{s}}} \left( {{\text{t}}^{\prime }} \right)} \right){\text{dt}}^{\prime } - {\text{data}}_{{{\text{t}}_{{{\text{ip}}}} }} } \right)^{2} ,\;{\text{and}} \\ \widehat{{{\uptheta }_{1} }} & = {\text{argmin}}\mathop \sum \limits_{{{\text{i}} = 1}}^{{\text{n}}} \left( {\mathop \smallint \limits_{{{\text{t}}_{{\text{i}}} }}^{{{\text{t}}_{{\text{i}}} + 1}} \left( {\left( {1 - {\uptau }_{{\text{s}}} } \right){{\updelta \uprho I}}_{{\text{s}}} \left( {{\text{t}}^{\prime }} \right) + {\upalpha {\text{L}}}_{{\text{m}}} \left( {{\text{t}}^{\prime }} \right)} \right){\text{dt}}^{\prime } - {\text{data}}_{{{\text{t}}_{{{\text{iq}}}} }} } \right)^{2} , \\ \end{aligned}$$

where ${\text{data}}_{{{\text{t}}_{{{\text{ip}}}} }}$ and ${\text{data}}_{{{\text{t}}_{{{\text{iq}}}} }}$ denote the DS and MDR-TB annual incidence data respectively and $\mathop \smallint \limits_{{{\text{t}}_{{\text{i}}} }}^{{{\text{t}}_{{\text{i}}} + 1}} \left( {\upalpha {\text{L}}_{{\text{s}}} \left( {{\text{t}}^{\prime }} \right)} \right){\text{dt}}^{\prime }$ and $\mathop \smallint \limits_{{{\text{t}}_{{\text{i}}} }}^{{{\text{t}}_{{\text{i}}} + 1}} \left( {\left( {1 - {\uptau }_{{\text{s}}} } \right){{\updelta \uprho I}}_{{\text{s}}} \left( {{\text{t}}^{\prime }} \right) + {\upalpha {\text{L}}}_{{\text{m}}} \left( {{\text{t}}^{\prime }} \right)} \right){\text{dt}}^{\prime }$ are the corresponding model solution at time ${\text{t}}_{{\text{i}}}$ respectively, while n is the number of available actual data points. The associated parameters of the model (2) are tabulated in Table 1. We assume the initial condition for the state variables are, ${\text{N}}\left( 0 \right) = 159,000,000$, ${\text{ I}}_{{\text{s}}} \left( 0 \right) = 205,899,{\text{L}}_{{\text{s}}} \left( 0 \right) = \frac{{{\upbeta {\text{I}}}_{{\text{s}}} \left( 0 \right){\text{N}}\left( 0 \right)}}{{\left( {{\upalpha } + {\upeta }_{{\text{s}}} + {\upmu }} \right)}} = 759,908,{\text{ I}}_{{\text{m}}} \left( 0 \right) = 1,100$, ${\text{ L}}_{{\text{m}}} \left( 0 \right) = \frac{{\left( {1 - {\text{c}}} \right){\upbeta {\text{I}}}_{{\text{m}}} \left( 0 \right){\text{N}}\left( 0 \right)}}{{\left( {{\upalpha } + {\upeta }_{{\text{m}}} + {\upmu }} \right)}} = 2,273, {\text{R}}\left( 0 \right) = 0{ }$ and ${\text{S}}\left( 0 \right) = {\text{N}}\left( {\text{0}} \right) - {\text{L}}_{{\text{s}}} \left( 0 \right) - {\text{I}}_{{\text{s}}} \left( 0 \right) - {\text{L}}_{{\text{m}}} \left( 0 \right) - {\text{I}}_{{\text{m}}} \left( 0 \right) - {\text{R}}\left( 0 \right)$.

**Table 1:** List of parameters, symbols, plausible values, units and references.

Description	Symbol	Value	Units	References
Bangladesh population in 2015	N	159,000,000		^12^
Bangladesh birth/death rate	µ	$\frac{1}{70}$	${\text{Year}}^{ - 1}$	^27^
Transmission rate	${\upbeta }$	$9.424 \times 10^{ - 8}$	${\text{Year}}^{ - 1}$	Fitted
MDR-TB fitness cost	c	0.45		Fitted
Progression rate from L to ${\text{I}}$	${\upalpha }$	0.40	${\text{Year}}^{ - 1}$	^28^
Natural recovery rate for DS-TB	${\upgamma }_{{\text{s}}}$	0.233	${\text{Year}}^{ - 1}$	^29^
Natural recovery rate for MDR-TB	${\upgamma }_{{\text{m}}}$	0.233	${\text{Year}}^{ - 1}$	^29^
TB related death rate	$\upphi$	0.39	${\text{Year}}^{ - 1}$	^29^
Treatment rate for DS-TB	${\uptau }_{{\text{s}}}$	0.94	${\text{Year}}^{ - 1}$	^30^
Treatment rate for MDR-TB	${\uptau }_{{\text{m}}}$	0.78	${\text{Year}}^{ - 1}$	^30^
Proportion of amplification	${\uprho }$	0.07		^31^
Rate of losing immunity	${\upomega }$	0.10	${\text{Year}}^{ - 1}$	^18^
Progression rate from ${\text{L}}_{{\text{s}}}$ to ${\text{S}}$	${\upeta }_{{\text{s}}}$	3.65	${\text{Year}}^{ - 1}$	^28^
Progression rate from ${\text{L}}_{{\text{m}}}$ to ${\text{S}}$	${\upeta }_{{\text{m}}}$	3.65	${\text{Year}}^{ - 1}$	^28^
Detection rate	${\updelta }$	0.87	${\text{Year}}^{ - 1}$	Default fitted to ensure proportion detected fits with WHO reported rates
Drug-susceptibility testing rate	${\upkappa }$	0.18	${\text{Year}}^{ - 1}$	^30^

### Calculation of the detection rate (${\varvec{\delta}}$)

**Figure 2:**
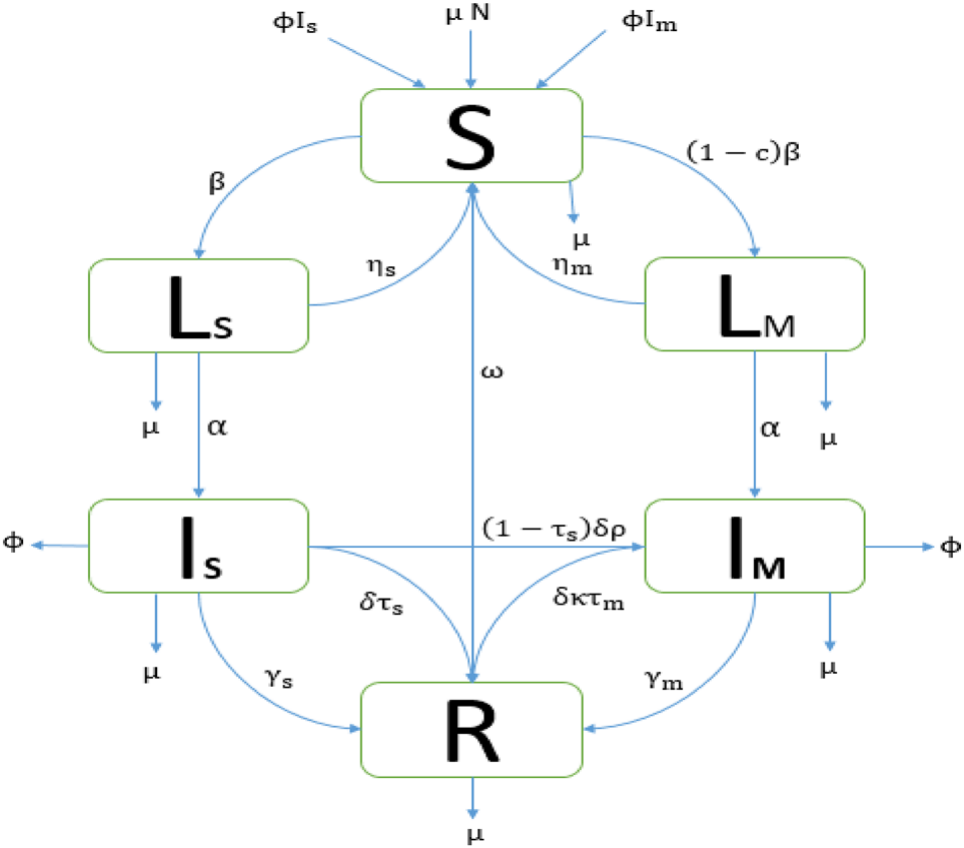
Schematic diagram of TB model for Bangladesh TB setting. System states and flow parameters are defined in the Methods section. Values for each of the model parameters can be found in Table 1.

We use the case detection proportion reported by^32^ to inform ${\updelta }$. This measure is often referred to as the “case detection rate” (${\text{CDR}}$) by WHO although it is actually a proportion. For the model presented in Fig. 2, the CDR is given by

$${\text{CDR}} = \frac{{\updelta }}{{{\upgamma }_{{\text{s}}} + \upphi + {\upmu } + {\updelta }}}$$

which we can rearrange to obtain.

$${\updelta } = \frac{{{\text{CDR}}}}{{\left( {1 - {\text{CDR}}} \right)}}{ }\left( {{\upgamma }_{{\text{s}}} + \upphi + {\upmu }} \right) = \frac{{0.578558011{ }}}{{\left( {1 - 0.578558011{ }} \right)}}{ }\left( {0.233 + 0.39 + \frac{1}{70}} \right) = 0.87,$$

where $${\text{CDR}} = \frac{{\text{Number of detected I}}}{{\text{Number of I}}} = \frac{209438}{{362000}}{ } = 0.578558011$$^32^.

### Sensitivity analysis

Sensitivity analysis is used to measure the degree of adequacy of our proposed model and determine which parameters impact on the model outputs^33,34^. Here, we considered the partial rank correlation coefficient (PRCC), a global sensitivity analysis metric to explore the influence of model parameters on the model outcomes^34,35^. To calculate the PRCC values, we employed the *Latin Hypercube Sampling* (LHS) technique (a stratified sampling without replacement technique which allows for an efficient analysis of parameter variation). Specifically, a uniform distribution is allocated from 0 to 3 times the baseline value for each model parameter and sampling is performed individually. A total of 1,000,000 simulations are implemented with sampled parameter values. In this analysis, the model outcomes we considered are the basic reproduction numbers ${\text{R}}_{{0{\text{s}}}}$ and ${\text{R}}_{{0{\text{m}}}}$.

**Figure 3:**
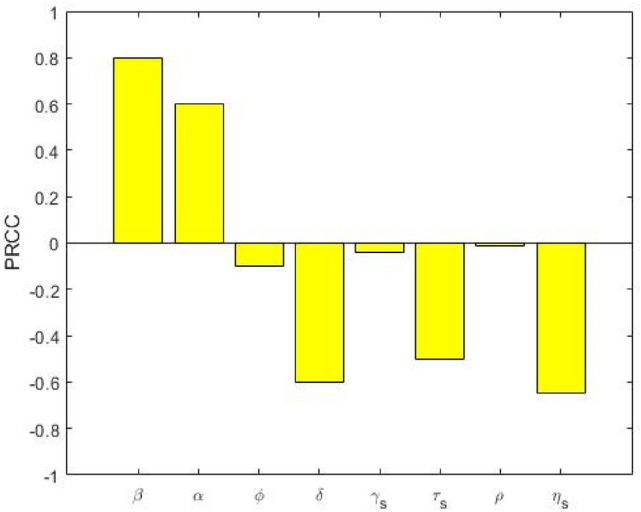
PRCC values depicting the sensitivity of the drug-susceptible basic reproduction number ${\text{R}}_{{0{\text{s}}}}$ with respect to the parameters ${\upbeta },{\upalpha },\upphi ,{\updelta },{\upgamma }_{{\text{s}}} ,{\uptau }_{{\text{s}}} ,{\uprho }$ and ${{ \upeta }}_{{\text{s}}}$.

**Figure 4:**
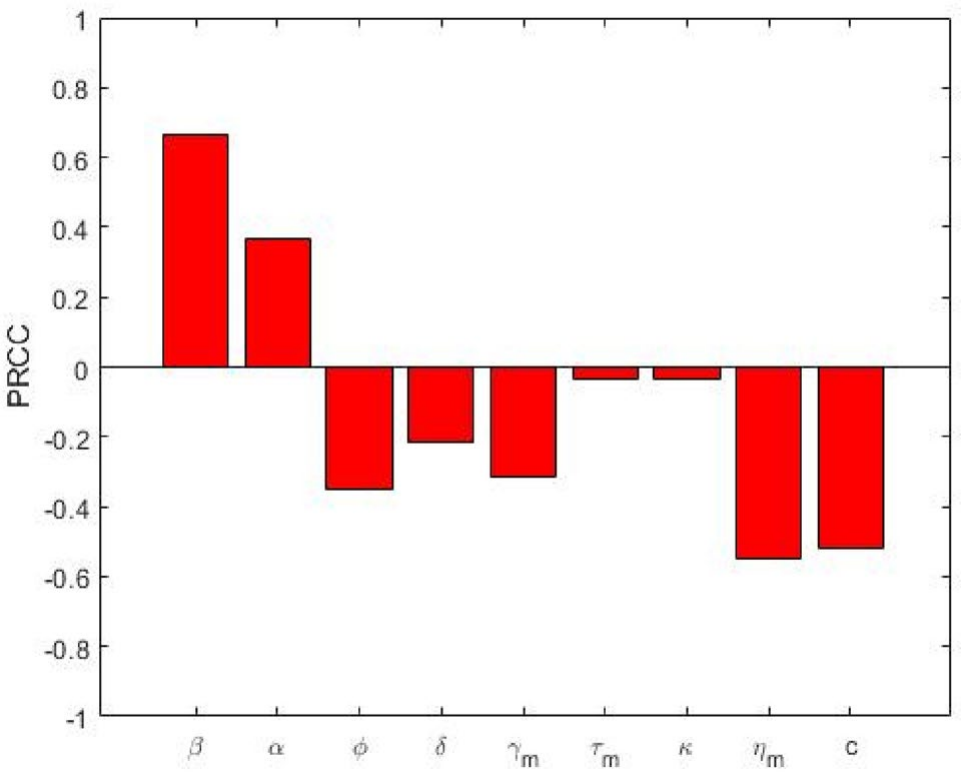
PRCC values depicting the sensitivity of the multidrug-resistant basic reproduction number ${\text{R}}_{{0{\text{m}}}}$ with respect to the parameters ${\upbeta },{\upalpha },\upphi ,{\updelta },{\upgamma }_{{\text{m}}} ,{\uptau }_{{\text{m}}} ,{\upkappa },{\upeta }_{{\text{m}}}$ and ${\text{c}}$.

Figures 3 and 4 depict the correlation between the basic reproduction numbers, namely ${\text{R}}_{{0{\text{s}}}}$ and ${\text{R}}_{{0{\text{m}}}}$, and the corresponding model parameters. Parameters ${\upbeta }$ and ${\upalpha }$ have positive PRCC values, implying that a positive change in these parameters (i.e. increasing transmission and progression rates) will increase the basic reproduction numbers ${\text{R}}_{{0{\text{s}}}}$ and ${\text{R}}_{{0{\text{m}}}}$. In contrast, parameters $\upphi,{\updelta },{\upgamma }_{{\text{s}}} ,{\uptau }_{{\text{s}}} ,{\uprho }$ and ${\upeta }_{{\text{s}}}$ have negative PRCC values with ${\text{R}}_{{0{\text{s}}}}$, which implies that raising these parameters will consequently decrease ${\text{ R}}_{{0{\text{s}}}}$. Further, parameters $\upphi ,{\updelta },{\upgamma }_{{\text{m}}} ,{\upkappa },{\uptau }_{{\text{m}}} ,{\upeta }_{{\text{m}}}$ and ${\text{c}}$ have negative PRCC values with ${\text{ R}}_{{0{\text{m}}}}$, which implies that increasing these parameters will consequently decrease ${\text{ R}}_{{0{\text{m}}}}$. We observed that the effect of the transmission rate ${\upbeta }$ has the largest influence on both ${\text{R}}_{{0{\text{s}}}}$ and ${\text{ R}}_{{0{\text{m}}}}$. Therefore, to control and eradicate DS and MDR-TB infection, it is important to minimize the transmission rate ${\upbeta }$.

From the explicit formulae for ${\text{R}}_{{0{\text{s}}}}$ and ${\text{R}}_{{0{\text{m}}}}$ given in equations (a)–(b), analytical expressions for the sensitivity indices to each of the parameters can be derived following the method in^36^. For example, for the parameter ${\upbeta }$ we have:

$${\Upsilon }_{{\upbeta }}^{{{\text{R}}_{{0{\text{s}}}} }} = { }\frac{{\partial {\text{R}}_{{0{\text{s}}}} }}{{\partial {\upbeta }}} \times \frac{{\upbeta }}{{{\text{R}}_{{0{\text{s}}}} }}.$$

Now using the parameter values in Table 1, we have the following results (Table 2).

**Table 2:** Sensitivity indices to parameters for the model (2).

Parameter	Sensitivity index (${\text{R}}_{{0{\text{s}}}}$)	Parameter	Sensitivity index (${\text{R}}_{{0{\text{m}}}}$)
${\upbeta }$	+ 1.000	${\upbeta }$	+ 1.000
${\upalpha }$	+ 0.902	${\upalpha }$	+ 0.902
$\upphi$	− 0.267	$\upphi$	− 0.514
${\updelta }$	− 0.647	${\updelta }$	− 0.161
${\upgamma }_{{\text{s}}}$	− 0.160	${\upgamma }_{{\text{m}}}$	− 0.307
${\uptau }_{{\text{s}}}$	− 0.521	${\uptau }_{{\text{m}}}$	− 0.161
${\uprho }$	− 0.003	${\upkappa }$	− 0.161
${\upeta }_{{\text{s}}}$	− 0.898	${\upeta }_{{\text{m}}}$	− 0.898
		${\text{c}}$	− 0.818

In the sensitivity indices of ${\text{R}}_{{0{\text{s}}}}$ and ${\text{ R}}_{{0{\text{m}}}}$, the most sensitive parameter is the effective contact rate $\left( {\upbeta } \right)$. Other significant parameters are the activation rate $\left( {\upalpha } \right)$, and progression rates $\left( {{\upeta }_{{\text{s}}} {\text{ and }}{{\upeta }}_{{\text{m}}} } \right)$, followed by fitness cost $\left( {\text{c}} \right)$. The least sensitive parameter is the amplification rate $\left( {\uprho } \right)$. Increasing (or decreasing) the effective contact rate, ${\upbeta }$ of DS-TB and MDR-TB by 100%, increases (or decreases) the reproduction numbers ${\text{R}}_{{0{\text{s}}}}$ and ${\text{R}}_{{0{\text{m}}}}$ by 100%. Similarly, increasing (or decreasing) the amplification rate $\left( {\uprho } \right)$ of DS-TB by 100% decreases (or increases)${\text{ R}}_{{0{\text{s}}}}$, by 0.3%.

### Scenario analysis

In this section, we analyzed multiple potential intervention scenarios. These scenarios are detailed in Tables 3 and 4. We parameterized these proposed responses to our model structure to assess the effect of these responses during the time period 2020–2035.

**Table 3:** Hypothetical single intervention strategy implemented in our proposed model of DS and MDR-TB control in Bangladesh, for the period 2020–2035.

Parameters	Parameter values	Estimated DS-TB annual incident cases	Reduction from baseline	Estimated MDR-TB annual incident cases	Reduction from baseline	Estimated DS-TB annual mortality	Reduction from baseline	Estimated MDR-TB annual mortality	Reduction from baseline
Proportion detected per year (CDR)	Baseline $\left( {58\% } \right)$	$7.35 \times 10^{6}$	$0.00 \times 10^{6}$	$1.26 \times 10^{6}$	$0.00 \times 10^{6}$	$3.15 \times 10^{6}$	$0.00 \times 10^{6}$	$0.65 \times 10^{6}$	$0.00 \times 10^{6}$
	$70\%$	$3.73 \times 10^{6}$	$3.62 \times 10^{6}$	$0.49 \times 10^{6}$	$0.77 \times 10^{6}$	$1.30 \times 10^{6}$	$1.85 \times 10^{6}$	$0.24 \times 10^{6}$	$0.41 \times 10^{6}$
	$80\%$	$0.34 \times 10^{6}$	$7.01 \times 10^{6}$	$0.08 \times 10^{6}$	$1.18 \times 10^{6}$	$0.92 \times 10^{4}$	$3.14 \times 10^{6}$	$0.04 \times 10^{6}$	$0.61 \times 10^{6}$
	$90\%$	$0.15 \times 10^{2}$	$7.35 \times 10^{6}$	$0.37 \times 10^{3}$	$1.26 \times 10^{6}$	$0.30 \times 10^{1}$	$3.15 \times 10^{6}$	$0.16 \times 10^{3}$	$0.65 \times 10^{6}$
DS-TB treatment success proportion	Baseline $\left( {94\% } \right)$	$7.35 \times 10^{6}$	$0.00 \times 10^{6}$	$1.261608 \times 10^{6}$	$0.00 \times 10^{6}$	$3.15 \times 10^{6}$	$0.00 \times 10^{6}$	$0.656690 \times 10^{6}$	$0.00 \times 10^{6}$
	$96\%$	$7.25 \times 10^{6}$	$0.10 \times 10^{6}$	$1.261610 \times 10^{6}$	$- 0.02 \times 10^{2}$	$3.09 \times 10^{6}$	$0.06 \times 10^{6}$	$0.646691 \times 10^{6}$	$- 0.1 \times 10^{1}$
	$98\%$	$7.16 \times 10^{6}$	$0.19 \times 10^{6}$	$1.261612 \times 10^{6}$	$- 0.04 \times 10^{2}$	$3.03 \times 10^{6}$	$0.12 \times 10^{6}$	$0.646692 \times 10^{6}$	$- 0.2 \times 10^{1}$
	$100\%$	$7.06 \times 10^{6}$	$0.29 \times 10^{6}$	$1.261614 \times 10^{6}$	$- 0.06 \times 10^{2}$	$2.96 \times 10^{6}$	$0.19 \times 10^{6}$	$0.646693 \times 10^{6}$	$- 0.3 \times 10^{1}$
MDR-TB treatment success proportion	Baseline $\left( {78\% } \right)$	$7.35 \times 10^{6}$	$0.00 \times 10^{6}$	$1.26 \times 10^{6}$	$0.00 \times 10^{6}$	$3.15 \times 10^{6}$	$0.00 \times 10^{6}$	$0.65 \times 10^{6}$	$0.00 \times 10^{6}$
	$85\%$	$7.36 \times 10^{6}$	$- 0.01 \times 10^{6}$	$1.13 \times 10^{6}$	$0.13 \times 10^{6}$	$3.16 \times 10^{6}$	$- 0.01 \times 10^{6}$	$0.57 \times 10^{6}$	$0.08 \times 10^{6}$
	$90\%$	$7.37 \times 10^{6}$	$- 0.02 \times 10^{6}$	$1.04 \times 10^{6}$	$0.22 \times 10^{6}$	$3.17 \times 10^{6}$	$- 0.02 \times 10^{6}$	$0.53 \times 10^{6}$	$0.12 \times 10^{6}$
	$95\%$	$7.38 \times 10^{6}$	$- 0.03 \times 10^{6}$	$0.95 \times 10^{6}$	$0.31 \times 10^{6}$	$3.17 \times 10^{6}$	$- 0.02 \times 10^{6}$	$0.48 \times 10^{6}$	$0.17 \times 10^{6}$
Proportion of frontline tests that have Drug-susceptibility testing $\left( {\upkappa } \right)$	Baseline $\left( {18\% } \right)$	$7.35 \times 10^{6}$	$0.00 \times 10^{6}$	$1.26 \times 10^{6}$	$0.00 \times 10^{6}$	$3.15 \times 10^{6}$	$0.00 \times 10^{6}$	$0.65 \times 10^{6}$	$0.00 \times 10^{6}$
	$50\%$	$7.49 \times 10^{6}$	$- 0.14 \times 10^{6}$	$0.11 \times 10^{6}$	$1.15 \times 10^{6}$	$3.21 \times 10^{6}$	$- 0.06 \times 10^{6}$	$0.05 \times 10^{6}$	$0.60 \times 10^{6}$
	$70\%$	$7.54 \times 10^{6}$	$- 0.19 \times 10^{6}$	$0.02 \times 10^{6}$	$1.24 \times 10^{6}$	$3.24 \times 10^{6}$	$- 0.09 \times 10^{6}$	$0.94 \times 10^{4}$	$0.64 \times 10^{6}$
	$100\%$	$7.58 \times 10^{6}$	$- 0.23 \times 10^{6}$	$0.17 \times 10^{4}$	$1.25 \times 10^{6}$	$3.26 \times 10^{6}$	$- 0.11 \times 10^{6}$	$0.73 \times 10^{3}$	$0.65 \times 10^{6}$

**Table 4:** Hypothetical combination intervention strategy implemented in our proposed model of DS and MDR-TB control in Bangladesh, for the period 2020–2035.

Scenarios	Parameters changed	Parameter values	Estimated DS-TB annual incident cases	Reduction from baseline	Estimated MDR-TB annual incident cases	Reduction from baseline	Estimated DS-TB annual mortality	Reduction from baseline	Estimated MDR-TB annual mortality	Reduction from baseline
Baseline	Proportion detected	58%	$7.35 \times 10^{6}$	$0.00 \times 10^{6}$	$1.26 \times 10^{6}$	$0.00 \times 10^{6}$	$3.15 \times 10^{6}$	$0.00 \times 10^{6}$	$0.65 \times 10^{6}$	$0.00 \times 10^{6}$
	DS-TB success	94%								
	MDR-TB success	78%								
	Genexpert use	18%								
Modest investment 1	Proportion detected	70%	$3.89 \times 10^{6}$	$3.46 \times 10^{6}$	$0.03 \times 10^{5}$	$1.25 \times 10^{6}$	$1.34 \times 10^{6}$	$1.81 \times 10^{6}$	$0.01 \times 10^{5}$	$0.64 \times 10^{6}$
	DS-TB success	96%								
	MDR-TB success	85%								
	Genexpert use	50%								
Modest investment 2	Proportion detected	80%	$0.34 \times 10^{6}$	$7.01 \times 10^{6}$	$0.003 \times 10^{1}$	$1.26 \times 10^{6}$	$0.09 \times 10^{6}$	$3.06 \times 10^{6}$	$0.001 \times 10^{1}$	$0.65 \times 10^{6}$
	DS-TB success	98%								
	MDR-TB success	90%								
	Genexpert use	70%								
Strong investment 5 years then revert to baseline	Proportion detected	90%	$0.26 \times 10^{6}$	$7.09 \times 10^{6}$	$0.002 \times 10^{1}$	$1.26 \times 10^{6}$	$0.08 \times 10^{6}$	$3.07 \times 10^{6}$	$0.000 \times 10^{1}$	$0.65 \times 10^{6}$
	DS-TB success	100%								
	MDR-TB success	95%								
	Genexpert use	100%								
Strong sustained investment	Proportion detected	90%	$0.69 \times 10^{1}$	$7.35 \times 10^{6}$	$0.000 \times 10^{1}$	$1.26 \times 10^{6}$	$0.15 \times 10^{1}$	$3.15 \times 10^{6}$	$0.000 \times 10^{1}$	$0.65 \times 10^{6}$
	DS-TB success	100%								
	MDR-TB success	95%								
	Genexpert use	100%								

**Figure 5:**
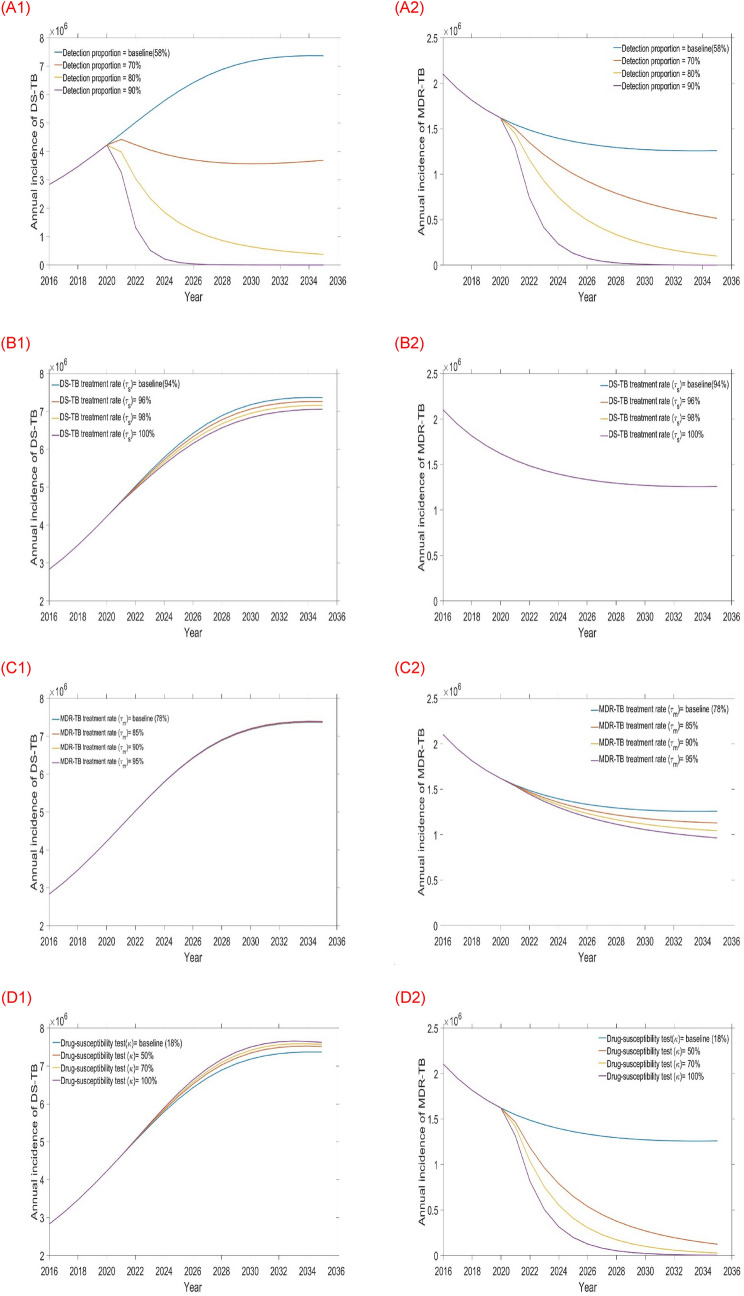
Impact of the four single intervention strategies on TB burden (left-hand side DS-TB annual incidence and right-hand side MDR-TB annual incidence): (**A1** and **A2**) varying detection rate, (**B1** and **B2**) varying DS-TB treatment rate, (**C1** and **C2**) varying MDR-TB treatment rate, and (**D1** and **D2**) varying Drug-susceptibility testing rate.

**Figure 6:**
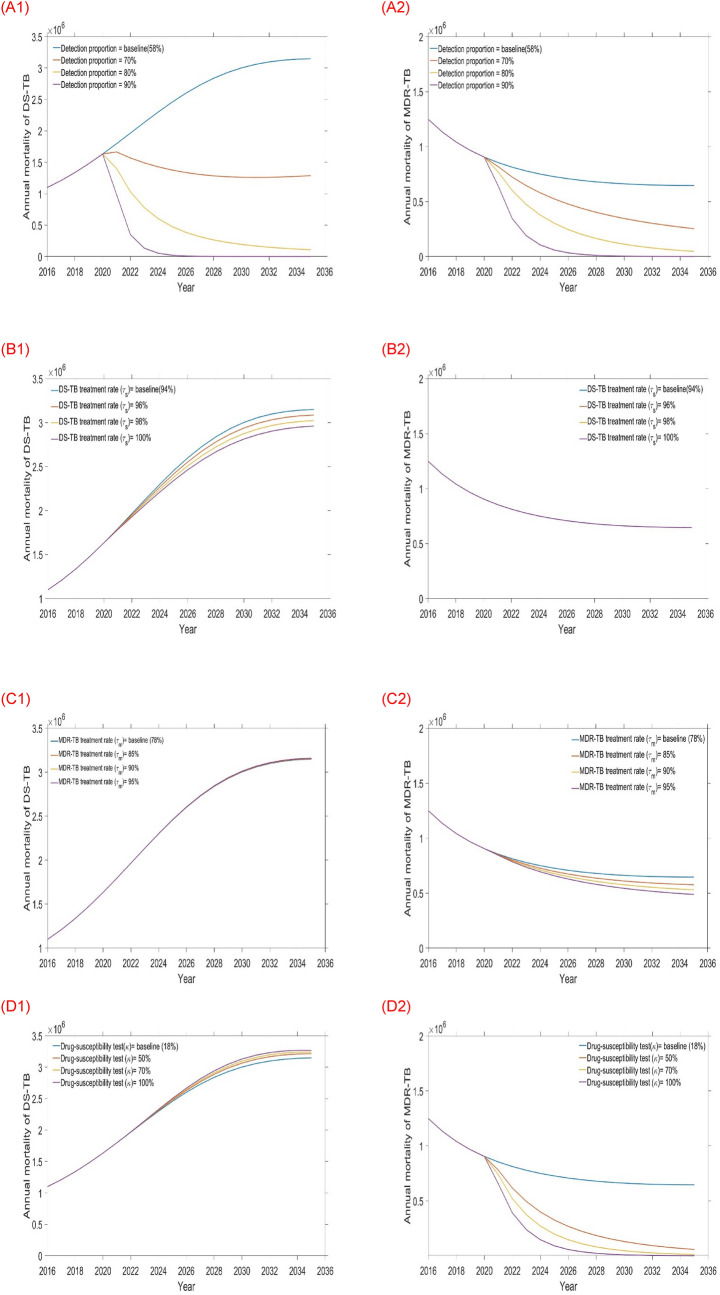
Impact of the four single intervention strategies on TB mortality (left-hand side DS-TB annual mortality and right-hand side MDR-TB annual mortality): (**A1** and **A2**) varying detection rate, (**B1** and **B2**) varying DS-TB treatment rate, (**C1** and **C2**) varying MDR-TB treatment rate, and (**D1** and **D2**) varying Drug-susceptibility testing rate.

Single intervention strategy simulates a continuation from the baseline values of each intervention to the high expected quantity of the programmatic situation during the time period 2020–2035. During this time, we simulated four separate intervention strategies: increasing the detection proportion (from baseline of 58% incrementally up to 90%); increasing both the DS and MDR-TB treatment rates (from baselines of 94–100% and from baseline of 78–95% respectively); and improving the drug-susceptibility testing rate (from 18% to 100%). We implement these as single interventions and compare them with baseline (see Table 3 and Figs. 5, 6) to explore the impact of each intervention on DS and MDR-TB incidence and mortality.

Results from the first tier of single intervention strategies are presented in Table 3, Figs. 5 and 6. From this tier, we observed that amongst the four single interventions considered increasing the detection proportion is more effective than any other single intervention at reducing DS and MDR-TB incidence and mortality (see Table 3 and Figs. 5A1,A2, 6A1,A2) in Bangladesh. Alternatively, the DS-TB treatment rate is another option for reducing DS-TB but has adverse impact on MDR-TB. Similarly, increasing the drug-susceptibility testing rate is additional choice for reducing MDR-TB.

**Figure 7:**
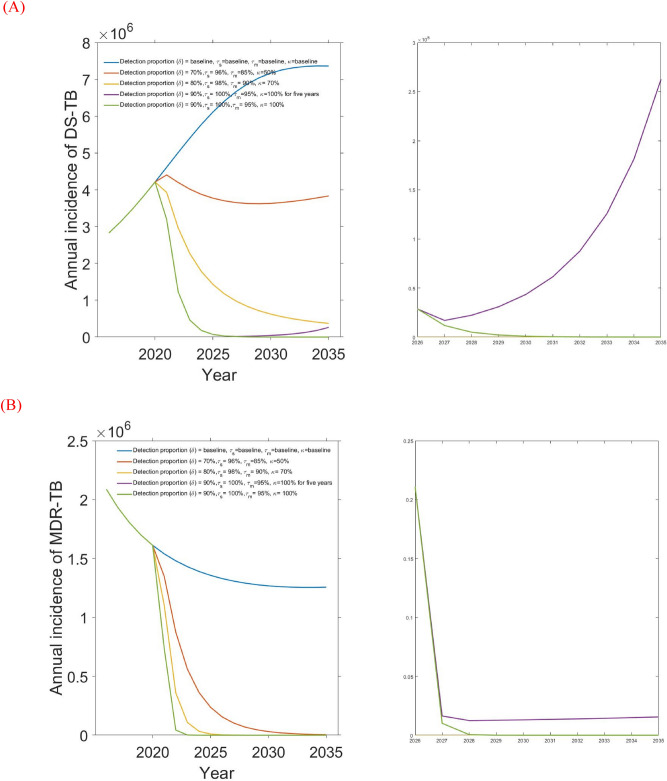
Combination intervention strategy and its effect on (**A**) DS-TB and (**B**) MDR-TB annual incidence in Bangladesh.

**Figure 8:**
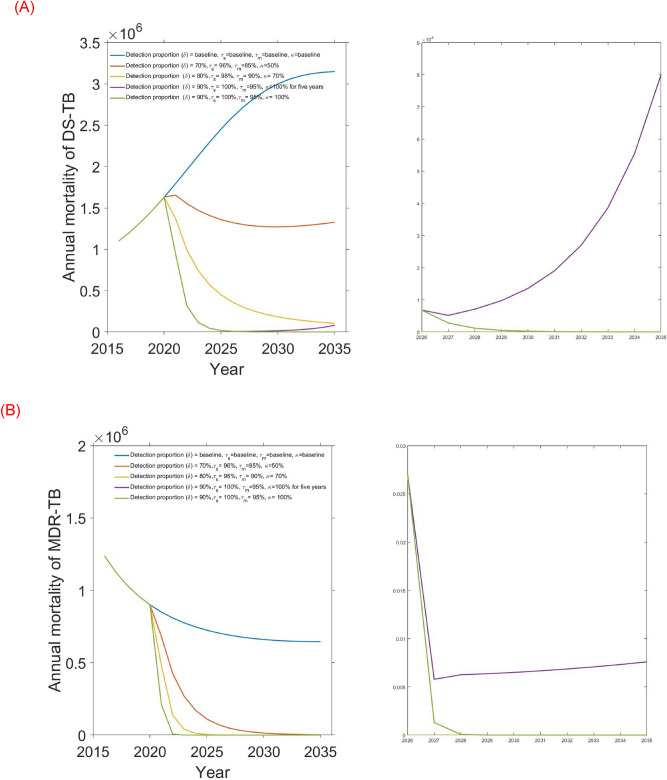
Combination intervention strategy and its effect on (**A**) DS-TB and (**B**) MDR-TB annual mortality in Bangladesh.

We next considered the combination of all four single-intervention strategies implemented simultaneously. Table 4, Figs. 7 and 8 present the outcomes for 5 combination strategies of incremental strength:

Baseline control strategy consists of a combination of baseline values of the four potential interventions including detection proportion (58%), DS and MBD-TB treatment rates (94% and 78%), and drug-susceptible testing rate (18%). The analysis shows that DS and MDR-TB are likely to increase with the current baseline control strategy.

Modest investment 1 intervention strategy which includes combination of detection proportion, DS and MBD-TB treatment rates, drug-susceptible testing rate from 58%, 94%, 78%, and 18% (baseline) to 70%, 96%, 85% and 50% respectively. AS expected, the strategy resulted in decreasing the number of DS and MDR-TB incidence and mortality in Bangladesh. Here, we observed that modest investment 1 strategy is most effective than the baseline strategy which reduce massive number of DS and MDR-TB incidence and mortality in Bangladesh (see Table 4, Figs. 7, 8). Modest investment 2 strategy represents combination of four potential interventions from baseline to 80%, 98%, 90% and 70% respectively. Result from this strategy shows that it is most successful than the modest investment 1 in light of not only reducing the number of DS and MDR-TB cases but also reducing the mortality.

The strategy strong investment 5 years and then revert to baseline includes extensive expansion of detection proportion, DS and MBD-TB treatment rates, drug-susceptible testing rate from baseline to 90%, 100%, 95% and 100% respectively for 5 years and then revert to baseline. The analysis shows that Strong investment for 5 years then revert to baseline strategy is highly effective for MDR with minimal rebound after reverting to baseline, as shown in Figs. 7 and 8. For DS-TB however, there is substantial rebound, but the strategy continues to out-perform the next best strategy (modest investment 2) up until 2035, as shown in Figs. 7 and 8.

Finally, Strong sustained investment strategy incorporates extensive expansion of detection proportion, DS and MBD-TB treatment rates, drug-susceptible testing rate from baseline to 90%, 100%, 95% and 100% respectively over 15 years period. The analysis shows that the strong sustained investment strategy is the most impactful intervention strategy, which reach the End TB targets reduces DS and MDR-TB cases by 90% and TB related death by 95% in Bangladesh. However, depending on funding availability, other scenarios in Table 4 can be considered.

## Discussion

Bangladesh is a resource poor, high burden TB country, and the transmission dynamics and epidemiology of TB are poorly understood. Recently, Bangladesh introduced programmatic management of MDR-TB at the community level to reduce the high utilization of inpatient beds that resulted from MDR-TB treatment under standard regimens^37^. As the effectiveness of the community-based short-course MDR-TB management Bangladesh regimen was found to be significantly higher than hospital-based management, it is important to identify the factors responsible for effective MDR-TB management, public awareness through education, and financial support from the treatment program, and programmatic strengths^12^. Although TB control in Bangladesh has significantly progressed—improved case finding, availability of free diagnostic and treatment services, involvement of multiple partners, newer diagnostic facilities, sufficient human resources, adequate capacity and guidelines—more effort is required.

In this paper, we presented a two-strain TB model with amplification: one strain for DS-TB; and another for MDR-TB. Here, we considered amplification as the process by which an individual infected with a DS-TB develops MDR-TB. We derived the basic reproduction number of each TB strain, and found that both basic reproduction numbers play an important role in the dynamics of DS and MDR-TB outbreaks. We fitted our model using DS and MDR-TB incidence data from the WHO Bangladesh reports. Sensitivity analyses were performed to determine the relative importance of several parameters used in our model. Our analysis led to the observations that, of the modifiable parameters, the *treatment of latent TB* parameter had the negative correlation with the basic reproduction numbers of DS and MDR-TB dynamics. This correlates with case detection (allowing increased treatment) having the greatest impact of the interventions and strongly suggests that investments in public health responses that focus on case detection should be the foundation of improved TB control.

In a previous TB modelling study^38^ we investigated the cost-effectiveness of time-varying combinations of different intervention strategies including distancing (which contains individual respiratory protection, environmental protection, diagnosis campaigns, and public awareness through education curricula), latent case finding (this contains chemoprophylaxis treatment, screening for high-risk exposure and additional procedures of latent TB treatment), case holding (this includes to actions that guarantee treatment completion to decrease relapse following treatment), and active case finding (this refers the prevention of disease progress with effective treatment for exposed individuals or identification of active TB cases) using an optimal control framework. This study found that for the single intervention strategy, the distancing control strategy is the most cost-effective for reducing the number of DS and MDR-TB cases in Bangladesh. However, the main finding of this study was that the combination of all intervention strategies is the most cost-effective.

In this paper we examine less-idealized strategies developed in partnership with the Bangladeshi National TB program. Specifically, we project the future outcomes of four specific intervention strategies: increased case detection proportion, improved drug-susceptibility testing and increased DS and MDR-TB treatment success, to assess the effect of these responses on our proposed TB model during the period from 2020 to 2035. Here, we additionally considered important parameters including the drug-susceptibility testing rate and reproductive fitness cost of MDR-TB that are not considered in the previous study^38^.

As a single-intervention, increasing the detection proportion was found to be the most effective strategy for reducing the incidence and mortality of DS and MDR-TB in Bangladesh compared to other single-intervention strategies, which is consistent with previous studies^25,26^. For DS-TB the second most effective intervention was improve treatment success rate, which reduces both mortality of the treated individual and transmission^1^. For MDR-TB, drug-susceptibility testing is the second most important intervention as it has a multiplicative effect with case detection in leading to proper implementation of effective second-line treatment^39^.

Traditional wisdom is that more treatment of drug-susceptible TB will translate to better outcomes for DS and MDR-TB. However, one condition in our study is that *increased* treatment does not mean *improved* treatment, so our model predicts that the risk of MDR acquisition for a given treatment course remains the same. If the MDR strain has reasonable fitness, such that the basic reproduction number is greater than one $({\text{R}}_{0} > 1)$, reducing the DS strain will increase the ecological niche for the resistant strain. Further, even if the MDR strain is not as fit as the DS strain, if treatment does not achieve and R__effective_ less than one for the DS strain, the MDR strain will increase through treatment with amplification. On the other hand, if ${\text{R}}_{0}$ is less than one for the MDR strain, then if treatment of DS-TB achieves and R__effective_ less than one, treatment (DOTS) for DS-TB will also eliminate MDR-TB.

We acknowledged the importance of comprehensive countrywide programmatic improvements to TB control in Bangladesh. Without such extensive approaches, further rises in the overall disease burden are expected, and the problem of drug resistance may possibly expand. Five scenarios all incorporating improved case notification, treatment success rates and drug susceptibility testing were examined to measure the effectiveness of these strategies.

From the analysis of implementing combination intervention strategies simultaneously, we found that a modest investment (detection proportion 70%, DS-TB treatment success 96%, MDR-TB treatment success 85%, drug-susceptibility testing 50%) is sufficient to substantially reduce MDR-TB, whereas a strong sustained investment strategy (detection proportion 90%, DS-TB treatment 100%, MDR-TB treatment 95%, drug-susceptibility testing 100%) is required to substantially reduce DS-TB. For MDR-TB, implementing multiple interventions simultaneously is the more effective than single intervention strategies, whereas for DS-TB which already has a very high treatment success rate in Bangladesh, case detection remains the key intervention.

Our strategies describe a variety of potential responses, extending from inaction to extremely ambitious multifactorial strategies. Despite the challenges faced in delivering effective programmatic TB control in Bangladesh, we believe it is essential to consider such responses because previous programs have demonstrated substantial public health gains in resource-limited settings such as Bangladesh^1^. Although the extensive approaches are not presently recommended by the World Health Organization or Bangladesh National TB Control Program, our modelling suggests that the high burden of DS and MDR-TB in Bangladesh is likely to increase with the existing, DOTS-based programmatic response.

## Methods and materials

### Model description

We developed a deterministic mathematical model of the transmission of DS and MDR-TB strains between the following mutually exclusive compartments: susceptibles ${\text{S}}\left( {\text{t}} \right)$, uninfected individuals who are susceptible to TB infection; those exposed to TB and latently infected ${\text{L}}\left( {\text{t}} \right)$, representing those who are infected and have not yet developed active TB; infectives ${\text{I}}\left( {\text{t}} \right)$, comprising individuals with active TB who are infectious; the recovered ${\text{R}}\left( {\text{t}} \right)$, who were previously infected and successfully cleared the infection through treatment or natural recovery. We use the subscripts s and m to denote DS-TB and MDR-TB quantities respectively.

The total population size, ${\text{N}}\left( {\text{t}} \right)$, is given by

1 $${\text{N}}\left( {\text{t}} \right) = {\text{S}}\left( {\text{t}} \right) + {\text{L}}_{{\text{s}}} \left( {\text{t}} \right) + {\text{I}}_{{\text{s}}} \left( {\text{t}} \right) + {\text{L}}_{{\text{m}}} \left( {\text{t}} \right) + {\text{I}}_{{\text{m}}} \left( {\text{t}} \right) + {\text{R}}\left( {\text{t}} \right) = {\text{N}}{.}$$

${\text{N}}$ is assumed to be constant and individuals mix randomly.

To assure the population size remains constant, we replace all deaths as newborns in the susceptible compartment. This involves death through natural causes, which occurs in all states at the constant per-capita rate ${\upmu }$, and TB-related deaths, which happen at the same constant per-capita rate $\upphi$ for individuals in the ${\text{I}}_{{\text{s}}}$ and ${\text{I}}_{{\text{m}}}$ compartments. Individuals may also return to the susceptible compartment following recovery at the constant per-capita rate ${\upomega }$. Individuals enter the susceptible compartment at a constant rate μ through birth$,$ where they may be infected with a circulating MTB strain at a time-dependent rate ${\uplambda }_{{\text{i}}} \left( {\text{t}} \right) = {\upbeta {\text{I}}}_{{\text{i}}} \left( {\text{t}} \right)$^38^. Here, ${\upbeta }$ is the probability of a susceptible individual being infected with MTB strain ${\text{i }}\left( {{\text{i}} = {\text{s}},{\text{m}}} \right)$ by an untreated infectious individual per day^38^. A proportion ${\upbeta {\text{I}}}_{{\text{s}}} \left( {\text{t}} \right){\text{S}}\left( {\text{t}} \right)$ and $\left( {1 - {\text{c}}} \right){\upbeta {\text{I}}}_{{\text{m}}} \left( {\text{t}} \right){\text{S}}\left( {\text{t}} \right)$ of the MTB susceptible individuals move to the latently infected compartment ${\text{L}}_{{\text{s}}} \left( {\text{t}} \right)$ and ${\text{L}}_{{\text{m}}} \left( {\text{t}} \right)$ respectively. Here, ${\text{c}}$ represents the MDR-TB fitness cost. We assumed that MDR-TB is initially generated through the inadequate treatment of DS-TB and could subsequently be transmitted to other individuals.

A proportion of latently infected individual’s progress to active TB as a result of endogenous reactivation of the latent bacilli at rates ${\upalpha }$. Moreover, since latently infected individuals have acquired partial immunity which reduces the risk of subsequent infection, a proportion also move to the susceptible compartment ${\text{S}}\left( {\text{t}} \right)$ at the constant per-capita rates ${\upeta }_{{\text{i}}} { }\left( {{\text{i}} = {\text{s}},{\text{m}}} \right)$. This rate can be accelerated by treatment of latent TB. Some infectious TB cases will undergo spontaneous recovery at a rate ${\upgamma }$, while others will die from TB-related causes at a rate,${{\upvarphi }}$. The remaining individuals with drug-sensitive and MDR active TB ${\text{I}}_{{\text{i}}} \left( {\text{t}} \right)$ will eventually be detected and treated at rates ${\updelta }$ and ${{ \uptau }}_{{\text{i}}} \left( {{\text{i}} = {\text{s}},{\text{m}}} \right)$ respectively. A proportion ${{\updelta \uptau }}_{{\text{s}}}$ of the treated DS active TB recover to move into the recovered compartment ${\text{ R}}\left( {\text{t}} \right),$ and a proportion of amplification $\left( {\uprho } \right)$ develops multi-drug resistance due to incomplete treatment or lack of strict compliance in the use of first-line drugs (drugs used to treat the DS forms of TB) to move into compartment ${\text{I}}_{{\text{m}}} \left( {\text{t}} \right)$.

To confirm MDR-TB we need to do extra drug-susceptibility testing $\left( {\upkappa } \right)$, therefore a proportion ${{\updelta \upkappa \uptau }}_{{\text{m}}}$ of MDR active TB cases recover to move to the recovered compartment ${\text{R}}\left( {\text{t}} \right)$. Furthermore, a proportion ${\upgamma }_{{\text{s}}} \;{\text{and}}\;{\upgamma }_{{\text{m}}}$ of individuals in compartments ${\text{I}}_{{\text{s}}} \left( {\text{t}} \right)$ and ${\text{I}}_{{\text{m}}} \left( {\text{t}} \right)$ naturally recover into ${\text{ R}}\left( {\text{t}} \right)$. A per-capita rate ${\upomega }$ from the recovered compartment ${\text{R}}\left( {\text{t}} \right)$ move into the completely susceptible compartment ${\text{S}}\left( {\text{t}} \right)$ due to the loss of immunity. The model flow diagram is presented in Fig. 2.

From the aforementioned, the system dynamics are governed by the following deterministic set of nonlinear ordinary differential equations:

2 $$\left\{ \begin{aligned} \frac{{{\text{dS}}}}{{{\text{dt}}}} & = {\upmu {\text{N}}} - {\upbeta {\text{I}}}_{{\text{s}}} {\text{S}} - \left( {1 - {\text{c}}} \right){\upbeta {\text{I}}}_{{\text{m}}} {\text{S}} - {\upmu {\text{S}}} + {\omega R} + \upphi {\text{ I}}_{{\text{s}}} + \upphi {\text{ I}}_{{\text{m}}} + {\upeta }_{{\text{s}}} {\text{L}}_{{\text{s}}} + {\upeta }_{{\text{m}}} {\text{L}}_{{\text{m}}} , \\ \frac{{{\text{dL}}_{{\text{s}}} }}{{{\text{dt}}}} & = {\upbeta {\text{I}}}_{{\text{s}}} {\text{S}} - {\upalpha {\text{L}}}_{{\text{s}}} - {\upmu {\text{L}}}_{{\text{s}}} - {\upeta }_{{\text{s}}} {\text{L}}_{{\text{s}}} ,{ } \\ \frac{{{\text{dI}}_{{\text{s}}} }}{{{\text{dt}}}} & = {\upalpha {\text{L}}}_{{\text{s}}} - {\upgamma }_{{\text{s}}} {\text{I}}_{{\text{s}}} - {\upmu {\text{l}}}_{{\text{s}}} - {{\updelta \uptau }}_{{\text{s}}} {\text{I}}_{{\text{s}}} - \upphi {\text{ I}}_{{\text{s}}} - \left( {1 - {\uptau }_{{\text{s}}} } \right){{\updelta \uprho I}}_{{\text{s}}} ,{ } \\ \frac{{{\text{dL}}_{{\text{m}}} }}{{{\text{dt}}}} & = \left( {1 - {\text{c}}} \right){\upbeta {\text{I}}}_{{\text{m}}} {\text{S}} - {\upalpha {\text{L}}}_{{\text{m}}} - {\upmu {\text{L}}}_{{\text{m}}} - {\upeta }_{{\text{m}}} {\text{L}}_{{\text{m}}} ,{ } \\ \frac{{{\text{dI}}_{{\text{m}}} }}{{{\text{dt}}}} & = {\upalpha {\text{L}}}_{{\text{m}}} - {\upgamma }_{{\text{m}}} {\text{I}}_{{\text{m}}} - {\upmu {\text{l}}}_{{\text{m}}} + \left( {1 - {\uptau }_{{\text{s}}} } \right){{\updelta \uprho I}}_{{\text{s}}} - \upphi {\text{ I}}_{{\text{m}}} - {{\updelta \upkappa \uptau }}_{{\text{m}}} {\text{I}}_{{\text{m}}} ,{ } \\ \frac{{{\text{dR}}}}{{{\text{dt}}}} & = {\upgamma }_{{\text{s}}} {\text{I}}_{{\text{s}}} + {\upgamma }_{{\text{m}}} {\text{I}}_{{\text{m}}} + {{\updelta \uptau }}_{{\text{s}}} {\text{I}}_{{\text{s}}} + {{\updelta \upkappa \uptau }}_{{\text{m}}} {\text{I}}_{{\text{m}}} - {\omega R} - {\upmu {\text{R}}}.{ } \\ \end{aligned} \right.$$

### Basic reproduction numbers

The basic reproduction number is well defined as the expected number of secondary cases created by a single infectious case introduced into a totally susceptible population. A disease can spread in a population only if the basic reproduction number is greater than one. An epidemic occurs when an infection spreads through and infects a significant proportion of a population. A disease-free population is possible when the basic reproduction number is less than one, which means that the disease naturally fades-out^40,41^. Here, we used the next-generation matrix method^42^ to estimate the basic reproduction numbers in our proposed model. The model has four infected states: ${\text{L}}_{{\text{s}}} ,{\text{I}}_{{\text{s}}} ,{\text{L}}_{{\text{m}}} ,{\text{I}}_{{\text{m}}}$, and two uninfected states: ${\text{S }}$ and ${\text{ R}}$. At the infection-free steady state ${\text{L}}_{{\text{s}}}^{0} = {\text{I}}_{{\text{s}}}^{0} = {\text{L}}_{{\text{m}}}^{0} = {\text{I}}_{{\text{m}}}^{0} = {\text{R}}^{0} = 0,$ hence ${\text{S}}^{0} = {\text{N}}.$ Since the total population size is constant, the only occurrence of the variable ${\text{S}}$ in equations $\left( {{\text{L}}_{{\text{s}}} ,{\text{I}}_{{\text{s}}} ,{\text{L}}_{{\text{m}}} ,{\text{I}}_{{\text{m}}} } \right)$, are either directly or implicitly via ${\text{N}}.$ To calculate the basic reproduction numbers of the DS and MDR-TB strains we follow^43^ and focus on the linearized infection subsystem derived from Eq. (2):

3 $$ \begin{aligned}\left\{ {\begin{array}{*{20}l} {\frac{{{\text{dL}}_{{\text{s}}} }}{{{\text{dt}}}} =\upbeta {\text{I}}_{{\text{s}}} S - {\uppsi }_{{\text{s}}} {\text{L}}_{{\text{s}}} ,} \vspace{5pt}\\ {\frac{{{\text{dI}}_{{\text{s}}} }}{{{\text{dt}}}} = \upalpha {\text{L}}_{{\text{s}}} - {\upchi }_{{\text{s}}} {\text{I}}_{{\text{s}}} ,} \vspace{5pt}\\ {\frac{{{\text{dL}}_{{\text{m}}} }}{{{\text{dt}}}} = \left( {1 - {\text{c}}} \right)\upbeta {\text{I}}_{{\text{m}}} S - {\uppsi }_{{\text{m}}} {\text{I}}_{{\text{m}}}, } \vspace{5pt}\\ {\frac{{{\text{dI}}_{{\text{m}}} }}{{{\text{dt}}}} = \upalpha {\text{L}}_{{\text{m}}} - {\upchi }_{{\text{m}}} {\text{I}}_{{\text{m}}} + \left( {1 - {\uptau }_{{\text{s}}} } \right)\updelta \uprho {\text{I}}_{{\text{s}}} } \\ \end{array} } \right.\end{aligned} $$

where $${\uppsi }_{{\text{s}}} = {\upalpha } + {\upmu } + {\upeta }_{{\text{s}}}$$, $${\uppsi }_{{\text{m}}} = {\upalpha } + {\upmu } + {\upeta }_{{\text{m}}}$$, $$\upchi _{{\text{s}}} =\upgamma _{{\text{s}}} +\upmu + \updelta\uptau _{{\text{s}}} +\upphi + \left( {1 -\uptau _{{\text{s}}} } \right){\updelta \uprho }$$ and $$\upchi _{{\text{m}}} =\upgamma _{{\text{m}}} +\upmu + \updelta \upkappa \uptau _{{\text{m}}} +\upphi .$$

We refer to the ODEs (3) as the infection subsystem, as it only describes the production of new infected individuals and changes in the states of existing infected individuals. If we set ${\mathbf{X}} = \left( {{\text{L}}_{{\text{s}}} ,{\text{I}}_{{\text{s}}} ,{\text{L}}_{{\text{m}}} ,{\text{I}}_{{\text{m}}} } \right)^{{\text{T}}}$, where T denotes transpose, we now want to write the infection subsystem in the form

4 $${\dot{\mathbf{X}}} = \left( {T + {\Sigma }} \right){\mathbf{X}}.$$

The matrix $T$ corresponds to transmissions and the matrix ${\Sigma }$ to transitions. They are obtained from system $\left( 3 \right)$ by separating the transmission events from other events: if we refer to the infected states with indices ${\text{i}}$ and ${\text{ j}}$, with ${\text{i}},{\text{j}} \in 1,2,3,4,{ }$ then entry $T_{ij}$ is the rate at which individuals in infected state ${\text{j}}$ give rise to individuals in infected state ${\text{i}}$ in the system. For the subsystem $\left( 3 \right)$ we obtain

$$T = \left( {\begin{array}{*{20}l} {\begin{array}{*{20}l} {{ }0} & {{\upbeta {\text{N}}}} \\ \end{array} } & 0 & 0 \\ {\begin{array}{*{20}l} 0 & 0 \\ \end{array} } & 0 & 0 \\ {\begin{array}{*{20}l} {\begin{array}{*{20}l} 0 \\ 0 \\ \end{array} } & {\begin{array}{*{20}l} 0 \\ 0 \\ \end{array} } \\ \end{array} } & {\begin{array}{*{20}l} 0 \\ 0 \\ \end{array} } & {\begin{array}{*{20}l} {\left( {1 - {\text{c}}} \right){\upbeta {\text{N}}}} \\ 0 \\ \end{array} } \\ \end{array} { }} \right)\;{\text{and}}\;{\Sigma } = \left( {\begin{array}{*{20}l} { - {\uppsi }_{{\text{s}}} } & 0 & 0 \\ {\upalpha } & { - {\upchi }_{{\text{s}}} } & 0 \\ {\begin{array}{*{20}l} 0 \\ 0 \\ \end{array} } & {\begin{array}{*{20}l} 0 \\ {\left( {1 - {\uptau }_{{\text{s}}} } \right){{\updelta \uprho }}} \\ \end{array} } & {\begin{array}{*{20}l} { - {\uppsi }_{{\text{m}}} } \\ {\upalpha } \\ \end{array} } \\ \end{array} { }\begin{array}{*{20}l} 0 \\ 0 \\ {\begin{array}{*{20}l} 0 \\ { - {\upchi }_{{\text{m}}} } \\ \end{array} } \\ \end{array} } \right).$$

Hence, the Next-Generation Matrix ${\text{K}}$ is four-dimensional, and given by (note the essential minus sign)

$${\text{K}} = - T{\Sigma }^{ - 1} = T\left( { - {\Sigma }^{ - 1} } \right),$$

$$\begin{aligned} & = \left( {\begin{array}{*{20}l} {\frac{{{\text{N}}{{\upalpha \upbeta }}}}{{{\uppsi }_{{\text{s}}} {\upchi }_{{\text{s}}} }}} & {\frac{{{{{\text{N}}\upbeta }}}}{{{\upchi }_{{\text{s}}} }}} & {\begin{array}{*{20}l} {0{ }} & 0 \\ \end{array} } \\ 0 & 0 & {\begin{array}{*{20}l} {0{ }} & 0 \\ \end{array} } \\ {\begin{array}{*{20}l} {\frac{{{{{\text{N}}\updelta \uprho }}\left( {{\text{c}} - 1} \right)\left( {{\uptau }_{{\text{s}}} - 1} \right)}}{{{\uppsi }_{{\text{s}}} {\upchi }_{{\text{s}}} {\upchi }_{{\text{m}}} }}} \\ 0 \\ \end{array} } & {\begin{array}{*{20}l} {\frac{{{{{\text{N}}\upbeta \updelta \uprho }}\left( {{\text{c}} - 1} \right)\left( {{\uptau }_{{\text{s}}} - 1} \right)}}{{{\upchi }_{{\text{s}}} {\upchi }_{{\text{m}}} }}} \\ 0 \\ \end{array} } & {\begin{array}{*{20}l} {\begin{array}{*{20}l} {\frac{{ - {{{\text{N}}\upalpha \upbeta }}\left( {{\text{c}} - 1} \right)}}{{{\uppsi }_{{\text{m}}} {\upchi }_{{\text{m}}} }}} & {\frac{{ - {\text{N}}{{\upbeta }}\left( {{\text{c}} - 1} \right)}}{{{\upchi }_{{\text{m}}} }}} \\ \end{array} } \\ {\begin{array}{*{20}l} {0{ }} & 0 \\ \end{array} } \\ \end{array} } \\ \end{array} { }} \right), \\ & = \left( {\begin{array}{*{20}l} {\text{A}} & {\text{B}} \\ {\text{C}} & {\text{D}} \\ \end{array} } \right) \\ \end{aligned}$$

where $${\text{A}} = \left( {\begin{array}{*{20}l} {\frac{{{{{\text{N}}\upalpha \upbeta }}}}{{{\uppsi }_{{\text{s}}} {\upchi }_{{\text{s}}} }}} & {\frac{{{{{\text{N}}\upbeta }}}}{{{\upchi }_{{\text{s}}} }}} \\ 0 & 0 \\ \end{array} } \right),{\text{B}} = \left( {\begin{array}{*{20}l} 0 & 0 \\ 0 & 0 \\ \end{array} } \right),{\text{C}} = \left( {\begin{array}{*{20}l} {\frac{{{\text{N}}{{\updelta \uprho }}\left( {{\text{c}} - 1} \right)\left( {{\uptau }_{{\text{s}}} - 1} \right)}}{{{\uppsi }_{{\text{s}}} {\upchi }_{{\text{s}}} {\upchi }_{{\text{m}}} }}} & {\frac{{{{{\text{N}}\upbeta \updelta \uprho }}\left( {{\text{c}} - 1} \right)\left( {{\uptau }_{{\text{s}}} - 1} \right)}}{{{\upchi }_{{\text{s}}} {\upchi }_{{\text{m}}} }}} \\ 0 & 0 \\ \end{array} } \right)$$ and

$${\text{D}} = \left( {\begin{array}{*{20}l} {\frac{{ - {\text{N}}{{\upalpha \upbeta }}\left( {{\text{c}} - 1} \right)}}{{{\uppsi }_{{\text{m}}} {\upchi }_{{\text{m}}} }}} & {\frac{{ - {\text{N}}{{\upbeta }}\left( {{\text{c}} - 1} \right)}}{{{\upchi }_{{\text{m}}} }}} \\ 0 & 0 \\ \end{array} } \right).$$

Now

$$\begin{aligned} \det \left( {\text{K}} \right) & = \det \left( {\text{A}} \right){\text{det}}\left( {{\text{D}} - {\text{CA}}^{ - 1} {\text{B}}} \right), \\ & = \det \left( {\text{A}} \right){\text{det}}\left( {\text{D}} \right). \\ \end{aligned}$$

The dominant eigenvalue of this matrix is equal to the basic reproduction number. In this system we have two dominant eigenvalues, one is for DS-TB and another is for MDR-TB, where

a $${\text{R}}_{{0{\text{s}}}} = \frac{{{{{\rm{N}}\upalpha \upbeta }}}}{{{\uppsi }_{{\text{s}}} {\upchi }_{{\text{s}}} }} = \frac{{{{{\text{N}} \upalpha \upbeta }}}}{{\left( {{\upalpha } + {\upmu } + {\upeta }_{{\text{s}}} } \right)\left( {{\upgamma }_{{\text{s}}} + {\upmu } + {{\updelta \uptau }}_{{\text{s}}} + \upphi + \left( {1 - {\uptau }_{{\text{s}}} } \right){{\updelta \uprho }}} \right)}}$$

and

b $${\text{R}}_{{0{\text{m}}}} = \frac{{{{{\text{N}}\upalpha \upbeta }}\left( {1 - {\text{c}}} \right)}}{{{{\uppsi }}_{{\text{m}}} {{\upchi }}_{{\text{m}}} }} = \frac{{{{{\text{N}} \upalpha \upbeta }}\left( {1 - {\text{c}}} \right)}}{{\left( {{{\upalpha }} + {{\upmu }} + {{\upeta }}_{{\text{m}}} } \right)\left( {{{\upgamma }}_{{\text{m}}} + {{\upmu }} + {{\updelta \upkappa \uptau }}_{{\text{m}}} + \upphi } \right)}}.$$

**Figure 9:**
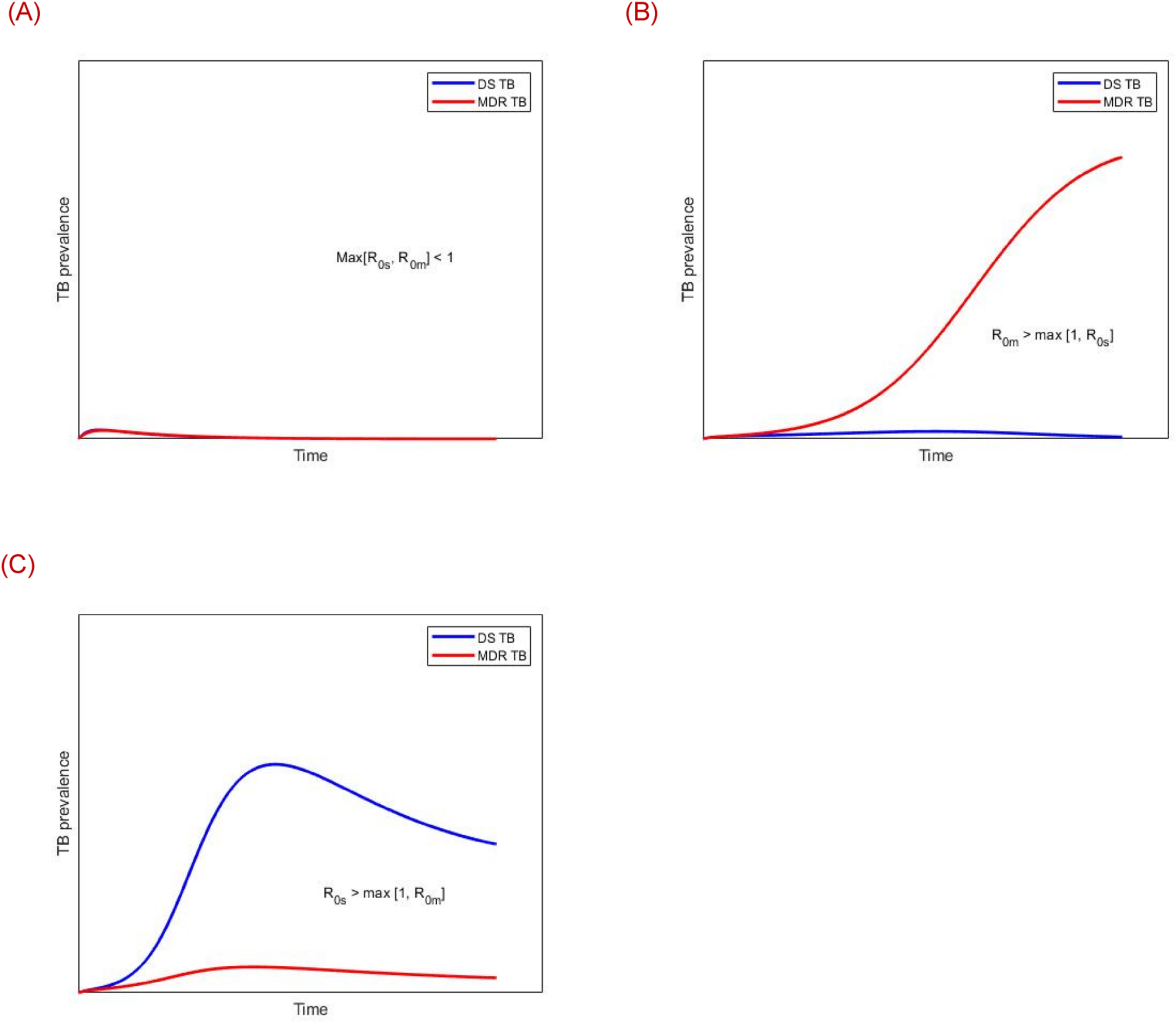
The effects of the strain-specific basic reproduction number on the dynamics of model (2).

The strain-specific reproduction numbers ${\text{R}}_{{0{\text{s}}}}$ and ${\text{R}}_{{0{\text{m}}}}$ regulate whether a particular strain will persist or fade out from the population in relation to the other strain. Figure 9A shows that TB disease will eventually die out from the population when the condition $\max \left[ {{\text{R}}_{{0{\text{s}}}} ,{\text{ R}}_{{0{\text{m}}}} } \right] < 1$ holds. The condition ${\text{R}}_{{0{\text{m}}}} > \max \left[ {{\text{R}}_{{0{\text{s}}}} ,{ }1} \right]$ implies that DS-TB dies out but MDR-TB persists in the population (see Fig. 9B). Finally, the condition ${\text{R}}_{{0{\text{s}}}} > \max \left[ {{\text{R}}_{{0{\text{m}}}} ,{ }1} \right]$ implies that both DS-TB and MDR-TB persist in the population (see Fig. 9C).

### Scenario development

In this section, we developed multiple potential intervention scenarios in consultation with staff at the National TB Control Program (NTP) in Bangladesh. The inputs parameters in this study, over a 15-year time frame, included: detection proportion, DS, and MDR-TB treatment rates, drug-susceptibility testing rate. Here, the detection proportion is improved through a combination of case finding strategies and improved knowledge of standard operating procedures for TB diagnosis and treatment commencement. Case finding is considered as identification of symptomatic patients attending a health facility, either of their own initiative or referred by another health facility, health worker, and community volunteer. These activities are assumed to progressively increase the detection proportion from baseline (58%) to 90%.

Further, DS and MDR-TB treatment rates are improved through infectious TB patients immediately seeking medical care and going to the health care facilities to undergo treatment. Programmatic management of drug-resistant TB (PMDT) is one of the most effective strategies for the control and prevention of DR-TB^44^. PMDT activities include proper management of contacts by ensuring that optimal treatment, a reliable drug supply and adequate health facilities are available^45^. Directly observed treatment, short-course (DOTS) is an important component in the internationally recommended policy package for TB control. During DOTS, a qualified practitioner observes the patient ingest their medication, which results in a demonstrable improvement in treatment rates and patient outcomes^30^. Accordingly, we assumed the DS and MDR-TB treatment rates progressively increased from (94% and 78%) to 100% and 95% successfully treated, respectively.

The efficient control of DS-TB and MDR-TB depends on rapid diagnosis, adequate and early initiation of treatment with the proper regimen, appropriate contact tracing, addressing adverse drug reaction and infection control measures in both facilities and communities. For diagnosis of MDR-TB, drug-susceptibility testing plays a vital role which is also recommended by global policy makers through the WHO’s End TB Strategy. In Bangladesh, around 18% of facilities are covered with drug-susceptibility testing by Gene Xpert^30^. Therefore, we assumed the drug-susceptibility testing rate, which includes the criteria for resistance, progressively increasing from baseline (18%) to 100% successfully tested. For each of these alternatives, the application of each separate intervention leads to a reduction in DS and MDR-TB incidence and mortality.

Each category of intervention could involve several potential specific activities. For example, DS and MDR-TB treatments could include training of doctors, nurse and pharmacists on TB guidelines, monitoring and managing of supplies of high quality drugs. Here, we considered different intervention scenarios including four-single, and their combination (baseline, modest investment 1, modest investment 2, strong investment 5 years then revert to baseline, and strong sustained investment) to assess the effect of these responses on DS and MDR-TB incidence and mortality during the time period from 2020 to 2035.

### Ethical approval

This study is based on aggregated TB surveillance data in Bangladesh provided by the World Health Organization. No confidential information was included because mathematical analyses were performed at the aggregate level. All of the methods were conducted under the approved research protocol. The research protocol was approved by the James Cook University human ethics approval board, H7300.

## Data Availability

The datasets produced during the study are available from the corresponding author on reasonable request.
